# Becoming Aware Through Internal Exploration: Understanding Psychotherapy on Conceptual and Neurobiological Levels

**DOI:** 10.1177/17456916251378430

**Published:** 2025-10-23

**Authors:** Nick Kabrel, Jaan Aru

**Affiliations:** 1 Department of Psychology, University of Zurich; 2 Digital Society Initiative, University of Zurich; 3 Institute of Computer Science, University of Tartu

**Keywords:** cognitive map, insight, mechanism, neuroscience, psychotherapy, therapeutic change

## Abstract

Becoming aware of previously unrecognized aspects of one’s psychological and behavioral challenges is one of the central mechanisms of positive psychotherapeutic change. Yet the specific neurocognitive processes that underlie new realizations remain poorly understood. What must occur in one’s mind and brain for awareness to emerge? Here, we present a novel, detailed, process-based framework for understanding how new awareness arises during psychotherapeutic dialogue. Central to this framework are the concepts of “mental navigation” and “cognitive map expansion,” which we explain at both the conceptual and neuroscientific levels. Namely, individuals construct internal world models in the form of cognitive maps. Mental-health difficulties may reflect maps that are overly rigid or narrow. Therapeutic change may thus involve expanding these maps by mentally navigating beyond their current boundaries and forming new trajectories in the conceptual and neural activity space. We conclude by exploring clinical-practice implications as well as offering directions for empirically validating this model.

Every now and then a man’s mind is stretched by a new idea or sensation, and never shrinks back to its former dimensions.

—Oliver Wendell Holmes, *The Autocrat of the Breakfast-Table*

It is now well established that psychotherapy is an effective evidence-based method for the treatment of a wide range of mental-health conditions. A number of meta-analyses and narrative reviews of rigorously controlled studies have demonstrated that various forms of psychotherapy work for children, adolescents, and adults (e.g., Berg & Høie, 2010; Campbell et al., 2013; Cuijpers et al., 2025a, 2025b; Harrer et al., 2025; Lambert & Ogles, 2004; Munder et al., 2019; Nathan & Gorman, 2007). More precisely, the efficacy and effectiveness of psychotherapy have been documented in several areas, including therapy for depression and anxiety (Barth et al., 2016; de Ponti et al., 2024; Papola et al., 2024; Plessen et al., 2023; Pompoli et al. 2018), posttraumatic stress disorder (Althobaiti et al., 2020; Cusack et al., 2016, Gerger et al., 2014; Gkintoni et al., 2024), substance use disorders (Dahlberg et al., 2022; Dellazizzo et al., 2023; McHugh et al., 2010), obsessive-compulsive disorder (Machado-Sousa et al., 2023; Olatunji et al., 2013), eating disorders (Godfrey et al., 2015; Monteleone & Abbate-Daga, 2024; Russell et al., 2023), bipolar disorder (Chatterton et al., 2017; Jones et al., 2023; Oud et al., 2016), suicidal behavior (Briggs et al., 2019; Calati & Courtet, 2016; van Ballegooijen et al., 2025), personality disorders (Cristea et al., 2017; Crotty et al., 2024; Katakis et al., 2023; Oud et al., 2018; Volkert et al., 2019), and many more.

However, despite its demonstrated effectiveness, the most crucial question persists: How does psychotherapy work? Current studies are primarily correlational, which means that researchers measure clients’ reports before and after treatment and observe that therapy has led to changes. What remains unclear, however, is exactly what occurs between the “before and after” that results in the changed mental state and leads us to see and perceive our lives and ourselves in a different way. To uncover this black box, a specific mechanism leading to therapeutic transformation should be demonstrated (Kazdin, 2007, 2009). Generally, a mechanism of transformation is an underlying process that explains how therapeutic interventions lead to symptom reduction or improvement (Kazdin, 2007). Change mechanisms are distinct from the specific techniques or interventions used in therapy. Specifically, they represent the “why” and “how” of therapeutic change, going beyond simply observing that change has occurred (Kazdin, 2007).

To date, there is no single, universally accepted explanation of how psychotherapy works across all approaches and orientations. Although numerous studies have been published on diverse mechanistic aspects of therapy (see, e.g., Lemmens et al., 2016, 2017; Marwood et al., 2018; Riess, 2011; Shahar et al., 2010; Tschacher & Meier, 2020; Zilcha-Mano et al., 2018, 2025), the question of which mechanisms are sufficient and necessary for change is still controversial.

Specifically, one of the main debates revolves around common and specific factors of psychotherapeutic change. Common factors are nonspecific factors that contribute to therapeutic change irrespective of the modality used to achieve it (Wampold & Imel, 2015). Examples of common factors include strong therapeutic alliance, empathy, and treatment expectations, among others. In contrast, specific mechanisms are assumed to be unique to particular therapies. For example, in the cognitive-behavioral tradition, emotional processing or new learning about threat and reward are often advanced as specific mechanisms of treatment (e.g., Powers et al., 2017), whereas in the psychodynamic tradition, this role belongs to transference (e.g., Høglend et al., 2008). A more balanced view consists of combining both (i.e., common and specific factors should work together to contribute to meaningful change). On the basis of this view, one could argue that common factors are needed to provide a general frame so that specific factors can be utilized effectively (Mulder et al., 2017). To illustrate, to facilitate effective cognitive and emotional processing (specific factors), therapeutic alliance as well as empathic support (common factors) might be necessary.

Still, the gap in our understanding of what precisely makes psychotherapy effective and what is sufficient poses a tough challenge and requires the development of a metaframework of therapeutic change that would be applicable to the majority of therapies, if not all of them. First, identifying key mechanisms could simplify and organize our understanding of numerous treatments. Second, comprehending how therapeutic transformation works might enable us to enhance these changes more effectively by developing diverse strategies to initiate essential change processes. Third, to ensure that the benefits of treatments are consistently translated from research to practical application, it is important to determine the necessary components of treatment that must be maintained. Last, the identification of robust mechanisms could advance psychotherapy to another level, rendering it a more mature science (Goldfried, 2019; Kazdin, 2009).

In pursuit of this aim, here we suggest a novel framework that we assume to be a metaframework applicable to any type of *dialogical* psychotherapy (i.e., therapies that primarily work through a conversation between two or more people, excluding, e.g., purely behavioral, art, or music therapies, in which a deep verbal analysis is not at the center of intervention). More precisely, we show that becoming aware is one of the most crucial mechanisms on which the majority of therapeutic modalities converge. Therefore, it is important to understand the intricacies of the process of becoming aware. In the attempt to do so, we introduce the concepts of “mental navigation” and “cognitive map expansion” that allow us to model the process of becoming aware across therapeutic modalities on both conceptual and neurobiological levels.

## Becoming Aware

If one wants to construct a metaframework of therapeutic change in dialogical therapies, one needs to identify indispensable processes applicable to every type of dialogical therapy. Therapeutic alliance or clients’ expectations are obvious candidates (Wampold & Imel, 2015). Yet the research on these topics is abundant in the literature. Hence, we concentrate on another, perhaps somewhat overlooked, feature of therapy—the process of becoming aware.

Becoming aware of the phenomena of one’s psychological life, including thoughts, emotions, behaviors, and more, is fundamental to psychotherapy. Notably, this process involves becoming aware of new information that was not present before or making novel connections between previously unconnected information. Arguably, one of the most familiar labels for this phenomenon is “insight” (Castonguay & Hill, 2007). However, insight often brings psychodynamic connotations, and that is why we use the more neutral label “becoming aware.” The process is also complex and sometimes cannot be captured by a single name or definition, as also highlighted by Hill et al. (2007). Therefore, we note that different labels are used in the literature to refer to diverse aspects of becoming aware. Examples include reflective functioning (Katznelson, 2014), self-understanding (Bell & Leite, 2016), self-knowledge (Carden et al., 2022), innovative moment (Gonçalves et al., 2009), decentering (Hayes-Skelton & Graham, 2013), metacognition (Wells, 2011), awareness enhancement (Goldfried, 2019; Gorlin & Békés, 2021), mindfulness (Segal et al., 2020), and probably many more. Each of these labels characterizes slightly different aspects, but the core goal is consistent: helping clients gain a clearer understanding and perspective of their own thoughts, feelings, actions, needs, and desires (Gorlin & Békés, 2021).

### Becoming aware as a common factor of therapeutic change

For more than a century, psychotherapy has operated on the principle that making unconscious aspects of an individual’s internal world conscious leads to positive therapeutic outcomes (Breuer & Freud, 1893). Despite the evolution of psychotherapeutic practices, this core principle remains largely unchanged to this day (see Table 1).

**Table 1.** Component of Becoming Aware Within Diverse Therapeutic Modalities

Therapeutic modality	Method	Outcome
Cognitive-behavioral therapy	Identify patterns of negative thinking, understand their underlying causes, and challenge and reframe these thoughts to promote more adaptive behaviors; conduct (imagined) exposure and behavioral experiments (Beck et al., 2024)	Increased awareness of the patterns underlying maladaptive thoughts and moods; experiential learning through the process of guided discovery during, e.g., cognitive reappraisal or belief modification
Psychodynamic psychotherapy	Understand how unconscious factors influence life through the exploration of past experiences, transference dynamics, and hidden conflicts (Shedler, 2010)	Increased awareness and self-understanding of the previously unconscious factors driving one’s thoughts and behavior
Narrative therapy	Achieve innovative moments by broadening one’s narrative to a more adaptive one, exploring alternative perspectives and co-constructing empowering life stories (Gonçalves et al., 2009)	Increased awareness of how the previous narrative has been driving one’s life and understanding of how it can be changed by incorporating broader explanations
Coherence therapy	Explicate underlying implicit schemas, find an adaptive alternative view, and identify discrepancies with current views (Ecker & Toomey, 2008)	Increased awareness of the implicit schema and awareness of its discrepancy from the actual world
Mindfulness-based therapy	Cultivate present-moment awareness through mindfulness practices and observe thoughts and emotions without judgment (Crane, 2017)	Increased awareness of internal experiences, emotions, physical sensations, and thoughts
Emotion-focused therapy	Access and externalize emotional responses to be better aware of how they function in different situations (Greenberg & Safran, 1989)	Increased awareness of emotional responses and their impact on behavior and relationships
Schema therapy	Identify and modify deeply ingrained maladaptive schemas through cognitive, behavioral, and experiential techniques (Young et al., 2006)	Increased awareness and understanding of maladaptive schemas and better emotional regulation

Note: As can be seen from this table, psychotherapy involves the emergence of awareness about some aspect of mental life, irrespective of the modality used to achieve it. Although this is just one possible sample of prominent approaches in psychotherapy showing how they lead to increased awareness, we believe the same principles could be abstracted from many more therapeutic modalities. Note that the methods and outcomes presented in this table are a simplified version of the actual principles to which these modalities adhere.

Becoming aware is generally considered a common factor of therapeutic change (Castonguay & Hill, 2007; Frank, 1961; Goldfried, 1980, 2019; Wampold et al., 2007) comparable to the therapeutic alliance in its impact on the outcomes of psychotherapy (Jennissen et al., 2018; see also Antichi & Giannini, 2023; Lane et al., 2022; Wampold et al., 2007). Indeed, a series of common factors models highlight the significant role of becoming aware. For example, one of the most influential common factors models (Goldfried, 1980, 2019) identifies five components, one of which is “facilitating client awareness of the factors associated with his or her difficulties” (Goldfried, 2019, p. 488). Wampold et al. (2007) also argued that therapeutic insight consists of constructing a functional understanding of one’s problems and acts as a beneficial common factor crucial to all psychotherapeutic modalities (see also Frank, 1961).

Another notable transtheoretical model of change (Prochaska & DiClemente, 1983; Prochaska & Norcross, 2001) outlines the psychological stages and corresponding therapeutic processes that clients experience during adaptive change. This model highlights the development of awareness, progressing from a precontemplative state to contemplation, insight, and, ultimately, implementation. Likewise, Stiles et al.’s (1990) influential model of assimilation incorporates different stages spanning from unconscious experiences to clarification, insight, and integration of the novel understanding into one’s internal model of the world, which results in the application of this knowledge in action and the eventual mastery of the problem.

Apart from theoretical models, consistent evidence also suggests that increased patient awareness positively correlates with and/or predicts treatment outcomes (Connolly Gibbons et al., 2007; Høglend & Hagtvet, 2019; Jennissen et al., 2018; Johansson et al., 2010; Kallestad et al., 2010; Tulver et al., 2023). For example, in a randomized controlled trial, Jennissen et al. (2021) found that early insight gains (baseline to Month 2) predicted subsequent depression reduction (Months 2–5) in dynamic therapy. In addition, Kallestad et al. (2010) found that insight near the end of therapy predicted improvement of symptom severity and interpersonal functioning during a 2-year follow-up period. In Connolly Gibbons et al.’s (2007) study, increased self-understanding was associated with reductions in depression symptoms and improvements in quality of life during treatment. Moreover, the study found that improvements in self-understanding from intake to termination significantly predicted reduced anxiety symptoms from termination to a 6-month follow-up, even when controlling for symptom changes during treatment. Høglend and Hagtvet (2019) showed that transference work significantly increased insight, which, alongside tolerance for affects, mediated improvements in interpersonal functioning over 4 years. In a naturalistic study, Castonguay et al. (2010) reported that self-awareness and self-insight were frequently cited as helpful events, with self-awareness being the most significant client-reported event. In addition, it was shown that the so-called innovative moments—instances when clients go beyond the problem-saturated narrative and construct a novel explanation—are conducive to therapeutic change (Gonçalves et al., 2009; Matos et al., 2009). Last, converging evidence from behavioral studies, neuroscience, and psychotherapy research suggests that being aware of the immediate consequences of one’s current behavior is crucial for motivating sustainable behavior change and is aligned with the reinforcement learning framework (Ludwig et al., 2020).

It should be noted, however, that becoming aware is obviously not a sole prerequisite of therapeutic change (Høglend & Hagtvet, 2019), and it may even be ineffective if the therapeutic alliance is weak, the realization does not align with the person’s motivation and goals, or a person does not take necessary actions postrealization (Kuncewicz et al., 2014). Therefore, becoming aware alone should not be considered a sufficient prerequisite for change (although we assume it could be the case for some people) but rather one of the most important factors that contribute to change. For such awareness to translate into lasting change, further components such as emotional processing, behavioral activation, and integration into the broader self-concept are often required. Moreover, these processes should be facilitated and stabilized through the therapeutic relationship or a broader supportive environment within a family or social circle.

To summarize, becoming aware is a crucial component of psychotherapy. Given this importance, we believe it is vital to understand (a) the process and mechanism leading to becoming aware and (b) how it can be facilitated by therapists in a real-world setting. Although this might seem like a fundamental goal of psychotherapy science, there is a notable lack of knowledge about how awareness comes about, both conceptually and neurobiologically. Therefore, in the following section, we introduce a novel framework to advance our understanding.

## A Novel Framework for Understanding the Process of Becoming Aware

### A missing component

Although most studies focus on the impact and role of awareness in achieving therapeutic outcomes, little attention is devoted to the specific process that should occur in one’s mind and brain for awareness to emerge.

**Fig. 1.**
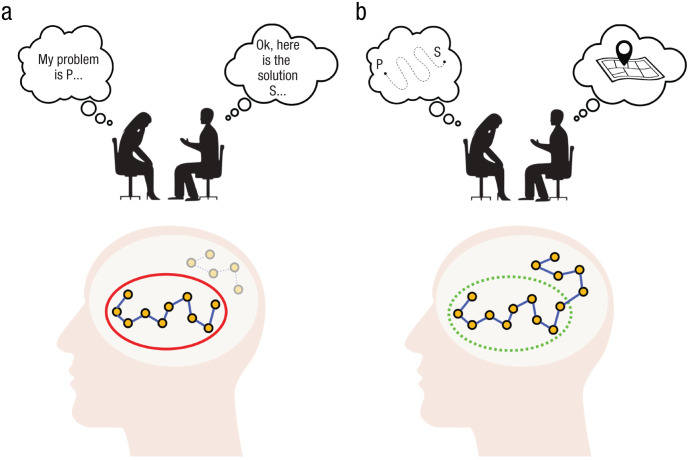
The importance of an internally activated mental navigation. Within our framework, if (a) the solution (S) to a problem (P) is presented to the client straightforwardly and without exploration, it cannot be integrated into the cognitive map and remains abstract and unattainable. Consequently, the cognitive map remains narrow (red circle), and the solution remains detached from this cognitive structure. When (b) the internal search is successful, meaning the therapist guides the client toward a novel understanding and the client actively explores and navigates toward it, the expansion of the cognitive map (dotted green circle) becomes possible.

To illustrate the importance of this process, let us consider a brief thought experiment. Imagine that a client explains their problem to a therapist and describes their symptoms and thoughts. The therapist then immediately offers an “insight” by explaining the root causes, reasons, and mechanisms of their issues and advising them on steps to solve the problem (see Fig. 1a). Would this kind of straightforward explanation instantly lead to a resolution of the problem?

Most researchers and practitioners would recognize that this is not how therapy works and not what is desired from it (see also Gendlin, 1969; Messer & McWilliams, 2007; Pascual-Leone & Greenberg, 2007; Wampold et al., 2007). Arguably, for awareness to emerge, some degree of personal elaboration or exploration is necessary (Messer & McWilliams, 2007). With this trivial thought experiment, we aim to make two points. First, for a psychotherapist to expand the client’s awareness, a prior exploration within the client’s mind must be conducted. This exploration is necessary for the therapist to learn the “cognitive map” of the client and understand how, when, and where to lead them to successfully expand their awareness. Second, even if there is a therapist who immediately offers a good “solution,” the client would arguably not be able to expand their awareness if the therapist straightforwardly provides it to them. External input alone is rarely sufficient for a client to meaningfully connect new information to their internal experiences. In this regard, we concur with Gendlin (1969), who argued that it is not a therapist or a specific method that facilitates change but something that successful clients do inside themselves.

Of course, this is not to imply that therapists should not provide any external information. Rather, therapists’ perspectives should always be accompanied by an exploration, from the client’s side, of how such information relates to their internal world. For example, in cognitive-behavioral therapy (CBT), there is a strong focus on guided discovery (e.g., Overholser & Beale, 2023), the core of which is rooted in an individualized case conceptualization and psychoeducation about the nature and interaction between triggers, emotions, thoughts, and behaviors (Kuyken et al., 2009). Such cases do not represent insights that clients derive on their own from their search process but rather general frameworks initially introduced by therapists. Yet, importantly, therapists then invite clients to work collaboratively to discover how the framework relates to their unique lives and experiences.

So, what should a successful client do internally to become aware of the underlying roots and reasons for a psychological problem? What is the qualitative step that distinguishes simply receiving the solution from coming to a realization on one’s own? In what follows, we argue that *mental navigation*, that is, an internally directed exploration process, must be present for the client to be able to embed the solution into their knowledge network.

### Cognitive maps

To understand the current framework, it is essential to grasp how individuals (a) store and structure information about the world in their minds (i.e., through the establishment of cognitive maps) and (b) further explore the contents of these cognitive maps (i.e., mental navigation). First, what are cognitive maps? In the mid-20th century, Tolman (1948) observed the fascinating ability of rats to remember the structures of mazes irrespective of the immediate reward. On the basis of these findings, he argued that we construct abstract and structured representations in the brain akin to maps. Therefore, “cognitive map” is a suitable term because it refers to structured mental as well as neural representations of life’s phenomena and their relations (e.g., physical, social, or conceptual space; Behrens et al., 2018; Bellmund et al., 2018; O’Keefe & Nadel, 1978; Tolman, 1948). Cognitive maps presumably house the representations of different objects, concepts, people, memories, and so on, as well as the relationships among them (e.g., Schiller et al., 2015). These mental representations are then used for flexible cognition and manipulation in working memory to make decisions, think, and solve problems (Behrens et al., 2018). These cognitive maps can be considered constituent parts of internal world models—a compressed, abstracted, and simplified representation of how the world works that allows individuals to understand, predict, and interact with their environment (Gärdenfors, 2000; Sutton & Barto, 1998; Wolpert et al., 1995; Yildirim & Paul, 2024).

An internal world model is thus a collection of diverse cognitive maps applicable to different contexts. The individual’s world model may be depicted as a large graph containing different interconnected concepts (Beaty & Kenett, 2023; Bieth et al., 2024; Herault et al., 2024; Kabrel et al., 2024; Siew et al., 2019) or as neural networks representing objects and relations between them (Behrens et al., 2018; Bellmund et al., 2018; Courellis et al., 2024; Morton & Preston, 2021; Neupane et al., 2024; Raju et al., 2024; Schiller et al., 2015). That said, brain correlates for cognitive maps have long been identified in the hippocampal-entorhinal system (see, e.g., Behrens et al., 2018; Bellmund et al., 2018; O’Keefe & Nadel, 1978).

It should be mentioned that cognitive maps and schemas are related constructs (Moscovitch et al., 2023; Young et al., 2006). They are not quite the same, however. Cognitive maps have well-characterized neural substrates in the hippocampal-entorhinal cortex, with mechanisms such as place cells, grid cells, and map-like coding of conceptual spaces (reviewed in Behrens et al., 2018; Bellmund et al., 2018). One can literally observe map-like coding in functional MRI (fMRI) and electrophysiological data (e.g., Constantinescu et al., 2016; S. A. Park et al., 2020, 2021; Viganò et al., 2021). Schemas in psychotherapy discourse, on the other hand, are most often treated as broader, more abstract cognitive structures (Beck et al., 2024; Young et al., 2006). Hence, research suggests that cognitive maps are a more precise, structurally grounded, and neurally plausible way of modeling what schemas describe at a higher level of abstraction (see also Behrens et al., 2018). In this regard, cognitive maps offer greater granularity: Whereas schemas represent overarching frameworks that shape cognition, cognitive maps can be thought of as a network composed of individual conceptual units that can be navigated one at a time within therapeutic dialogue. That said, a further reason for favoring the term “cognitive map” is its grounding in research on mental navigation (Courellis et al., 2024; Neupane et al., 2024; Viganò & Piazza, 2020), which has been shown to operate within cognitive maps specifically, not schemas. Last, the cognitive map and its expansion via navigation might serve as a powerful conceptual metaphor for understanding the crucial detail of the process of becoming aware.

### Mental navigation

The brain encodes knowledge in structured cognitive maps not only to effectively reflect the environment (Tolman, 1948) but also to enable the orientation and exploration of the contents of the cognitive maps for adaptive problem-solving and decision-making (Aru et al., 2023; Bellmund et al., 2018; Behrens et al., 2018; Neupane et al., 2024). We suggest using the term “mental navigation” to model this process (see also Aru et al., 2023; Kabrel et al., 2024; Ward & Plagnol, 2019). This framework is based on recent findings from the cognitive neuroscience of navigation that suggest a similar computational neural mechanism between physical territory navigation and the navigation of mental contents (Bellmund et al., 2018; Behrens et al., 2018; Raju et al., 2024). Thus, mental navigation is our ability to move through the contents of our cognitive maps, whether these maps represent physical spaces or, for instance, the structure of a problem (Aru et al., 2023; Behrens et al., 2018; Bellmund et al., 2018; Kabrel et al., 2024). Everything that we can direct attention to becomes a potential target for navigation (i.e., not only vivid mental images but also bodily feelings, intentions, and even more vague phenomenological experiences, such as when one wants to eat but struggles to see exactly what it is they want to eat). Mental navigation can be effortful and conscious or influenced by unconscious processes, such as during mind wandering. We can direct our attention to external stimuli (e.g., sounds in a room) or internal states (e.g., how hungry one is on a scale from 1 to 5).

There is a set of experiments that study how mental navigation works in different information spaces (see Todd et al., 2012). The outcomes of this research suggest the continuity between search behavior in spatial environments, which involves foraging for resources, and internal information search, that is, looking through cognitive representations to recall and reconstruct information from memory (e.g., Hills et al., 2008; Todd & Hills, 2020). This suggests that searching, whether it involves physical movement or not, requires navigating through some form of space in pursuit of resources. In other words, an individual must decide whether to move (either physically or mentally, i.e., by redeploying their attention) or stand still, and if they choose to move, they must determine the direction (Hills & Dukas, 2012).

In what follows, we show that mental navigation might serve as a powerful concept for understanding the process of psychotherapy. First, self-exploration in psychotherapy can be conceptualized as mental navigation within a given problem space (Kabrel et al., 2024). Second, as individuals are often unaware of the contents of their cognitive maps, engaging in mental navigation might contribute to enhanced awareness about these contents. Third, there might be problematic patterns of mental navigation (e.g., too rigid or repetitive thoughts, tendency to “go around in circles,” jumping to conclusions). Fourth, and perhaps most important, psychotherapy provides the opportunity to transform one’s internal space. This may be possible either by expanding the map (i.e., introducing new and meaningful representations) or by shifting how one navigates it (i.e., reaccessing, reweighting, or exploring previously ignored aspects of experience). Although below we place a primary focus on the expansion of the map, we acknowledge that therapeutic progress may also result from changes in navigation patterns.

### Narrow cognitive maps

Cognitive maps provide us with the remarkable ability to quickly learn and generalize knowledge to new situations we have never encountered before (e.g., Behrens et al., 2018; Courellis et al., 2024). For instance, if a mammal encounters a tiger in a particular territory, it learns to be cautious when approaching the same area in the future (Suddendorf & Redshaw, 2022). However, in the modern world, despite being evolutionarily useful, our ability to construct cognitive maps can sometimes have a reverse effect. For example, if one assumes that “people are hostile”—a conviction based on early relationships—they could extend this assumption to a broad array of people without understanding that different people might behave in different ways in different situations.

The central idea is that our cognitive maps are limited by the amount and quality of mental representations available to us for thinking about certain issues. To take an analogy, in the same way that a geographical map can be outdated or misrepresent a territory, our cognitive maps can also become too rigid or narrow, leading us to experience significant distress (see also Kabrel et al., 2024).

As we showed above, cognitive maps are based on previous experiences and have a certain structure. It is difficult for a person to abandon this internal structure; one could even say that people are “trapped” within their cognitive map, meaning that they cannot always simply go beyond it. Although people might realize that their understanding is limited, most often they have no clue about the limits of their understanding. A prime example of this would be depression, which is characterized by high cognitive rigidity or biases, rumination, overgeneralization, and other related features (Phillips et al., 2010). For instance, a depressed person, especially if depression is recurrent, may have negative self-referential thoughts and a tendency to come back to them even after an intervention directed at belief modification (Hayes & Andrews, 2020). Moreover, persistent implicit cognitive biases in depression may lead a person to interpret different situations through the same negative lens (Phillips et al., 2010).

When cognitive maps are established, we tend to continue navigating the preestablished cognitive (or neural) pathways to explain life’s phenomena or people’s behavior (Kahneman, 2011; McNally & Jean-Richard-dit-Bressel, 2024; Phillips et al., 2010; Saez et al., 2014). This tendency is documented in different studies on cognitive heuristics (Kahneman, 2011), for example, and by diverse concepts, such as functional fixedness (Duncker, 1945) or the Einstellung effect (Luchins, 1942). These concepts highlight how people often struggle to use objects or ideas in novel ways because their cognitive maps are too rigidly defined by past experiences. When thinking of some issue, we might not even realize that we are navigating along a preestablished, restricted trajectory, lacking the opportunity to voluntarily change the direction or the scope of our thinking (Duncker, 1945; Luchins, 1951; Saez et al., 2014). For example, research on metacognitive overconfidence shows that people might be blindly confident in some information or skill, whereas in reality it is wrong or incorrect (Fleming, 2024). Once we have encoded the trajectory, either a spatial or a mental one, we tend to exploit it in the future (Kahneman, 2011; McNally & Jean-Richard-dit-Bressel, 2024; Phillips et al., 2010; Saez et al., 2014).

This issue becomes even more pronounced when dealing with mental health problems. In this case, to say that our cognitive maps are limited because of a lack of knowledge or ignorance is insufficient. For example, Smith et al. (2020) illustrated that when we learn certain strategies to fulfill our needs, these strategies are reinforced every time the need is fulfilled successfully. A crucial observation in psychotherapy is that people can sometimes meet their needs through maladaptive behaviors. For instance, a child might feel safe in the presence of a mother; however, the mother behaves in a hostile and controlling way. In this way, the child learns a rule that to fulfill a need for safety they need to stick to a hostile, controlling person, thus choosing corresponding partners in their adult life.

Hence, a cognitive map is structured to serve a particular need or goal. This explains why presenting an individual with a solution or alternative behavior might not resonate with them (Fig. 1a). It is not simply a matter of ignorance (Chamberlin, 2023): It is because their current cognitive map serves a specific purpose. This is precisely why a person must become aware of the existing pattern and be internally guided on how to fulfill their needs differently (Fig. 1b). For this reason, we argue below that becoming aware can be fruitfully conceptualized as mentally navigating beyond the narrow cognitive map with the external help of a skilled guide—a psychotherapist.

### The expansion of the cognitive map

Relying on a previous experience too much can be seen as overly exploiting preexisting trajectories while not being aware of other, more adaptive ones. In essence, it could be seen as people mentally navigating around in circles, using the most exploited connections, and failing to discover shortcuts or more effective pathways to solve their problems (Kabrel et al., 2024; Smith et al., 2020). Hence, for a cognitive shift, novel connections and trajectories must be developed, restructured, and augmented; that is, the cognitive map must be expanded. In keeping with the metaphor, the expansion of the cognitive map through mental navigation can be seen as analogous to the expansion of a geographical map when new territories are discovered through physical navigation.

**Fig. 2.**
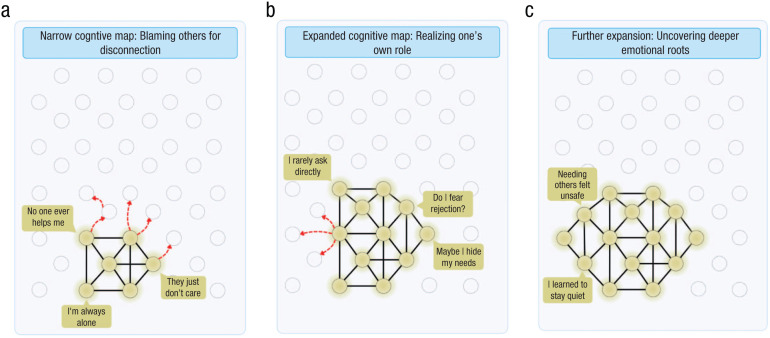
Adaptive expansion of a navigable territory within conceptual and neural state spaces. A person navigating within (a) the narrow cognitive map conceptualizes their problem as hopeless and blames others’ behavior as the reason for their discomfort. The shining nodes represent the activation of the network, and the transparent nodes are potential or hypothetical states that remain unexplored. The red dotted lines represent the therapist’s questions leading the client to navigate the unexplored space. In (b) the adaptive expansion of the navigable space, the therapist guides the client in discovering and mapping out new places that reflect a broader view: It is not only about others’ behavior but also how the client’s own behavior contributes to this problem. The (c) iterative expansion of the cognitive map shows that the client can go even deeper and explore the root causes. Note that there is no substitution of maladaptive thoughts with more adaptive ones (such as in belief modification) but rather constantly realizing more about one’s own thinking and behavioral patterns.

Expanding one’s cognitive map means mentally navigating beyond one’s habitual trajectories for thinking about or explaining a problem (Fig. 2) so that new trajectories become available to the person in the conceptual as well as neural activity space. These new mental areas become “mapped” and can be revisited—something that was not possible before. If previously a person’s habitual trajectory entailed a path of minimal resistance, with a more expanded repertoire, that person can choose a new direction with more agency and choice.

However, although expanding the map is the main focus of this article, it is not the only possibility for therapeutic change. Specifically, the issue may not lie in the narrowness of the map itself but rather in how individuals navigate it, that is, in how they direct their attention (see also Hills, 2024). Therefore, we view these two possibilities as complementary pathways contributing to change. To illustrate, we propose that in some cases the map may already contain all the necessary elements for understanding the problem, but some of these elements are habitually ignored or underexplored. In such cases, therapeutic change does not require expanding the map by introducing entirely new insights or concepts. Instead, the therapist helps the client redirect attention toward already known, yet less preferred or less frequently accessed, parts of the map. This type of mental navigation resembles belief-modification techniques, in which the goal is to shift the client’s focus from dominant negative thought patterns to alternative, often more adaptive, perspectives (D. A. Clark, 2014; Ellis, 1994). For instance, clients who habitually describe themselves as a failure might be asked to recall three instances of success. This activates alternative nodes in the map not by adding new content but by revisiting and reweighting existing content (see also Moscovitch et al., 2023). In a sense, it is still a form of expansion—not of the cognitive map itself but of the navigational patterns within it.

Note, however, that in our framework, the expansion of the cognitive map is not a mere restatement of therapeutic improvement but a hypothesized internal process through which change becomes possible. That is, the outcome of therapy (e.g., reduced symptoms, greater behavioral flexibility, enhanced well-being) is not identical to this expansion but may result from it through a variety of downstream processes such as behavioral experimentation, emotion regulation, or narrative integration. We therefore hypothesize that cognitive map expansion acts as a mediating mechanism that may influence other important variables such as increased self-awareness, greater behavioral variance, and improved emotion regulation. Importantly, not every case of therapeutic success entails cognitive map expansion, and not every expansion necessarily leads to immediate therapeutic gains. Rather, expansion is conceptualized as one distinct component within the overall change process. We return to this point later by outlining testable hypotheses derived from this framework. The next important question is how such an expansion is achieved.

#### Internally directed exploration

Although mental navigation might be performed on an externally directed task (e.g., solving a nine-dot problem), mental navigation in activities such as psychotherapy entails an internally driven exploration process. This means that a person directs their attention inwardly, to the contents of their internal world, and navigates within. As we pointed out earlier, psychotherapy clients are often prone to navigate among the well-established pathways. Therefore, we view the crucial role of the therapist in guiding the direction of clients’ mental navigation to those places that are hidden from them or the places that the client intentionally avoids (i.e., beyond the boundaries of their habitual navigational trajectories; Kabrel et al., 2024). As noted, such navigation can entail two distinct but highly intertwined pathways: one through structural expansion (i.e., expanding the number of available mental representations) and another through expanding the navigational patterns to include already mapped but rarely accessed representations. This means that the therapist should be able to detect the problematic (or narrow) patterns within the client’s navigation process and try to redirect their attention to the “unexplored territory,” often entailing a broader view of the problem. For example, a CBT-oriented therapist who detects a negative self-referential bias would try to redirect the client’s attention to consider a broader picture by using guided discovery or Socratic questioning (Overholser & Beale, 2023).

A vivid analogy can be made with an inexperienced person tasting red wine. Being unfamiliar with the breadth of wine flavors, the person is unable to perceive them at first. Therefore, for them, every wine has simple and similar tastes. However, if a seasoned sommelier directs the attention of the novice to explore and consciously attend to the array of tastes the wine contains, they will begin to perceive the wine taste differently, even though the wine itself has not changed (Pascual-Leone & Greenberg, 2007). Similarly, one could argue that the majority of people with mental-health problems are unaware of the structure of their cognitive map or the maladaptive rules and convictions they unconsciously follow (see also Dimaggio, 2015). The advantage of psychotherapeutic work is that, with the help of psychotherapists, clients are able to pave the way for their conscious attention to “reach” previously hidden places that influence their lives. By further working on these places, clients incorporate them into their cognitive landscape. This incorporation of previously inaccessible information allows one to “unlock” broader possibilities to apply in different situations and ultimately change the perception of the problem (see also Ludwig et al., 2020).

#### Conceptualization from experience

Whereas the therapist’s role may be in directing the client to arrive at a novel realization, clients themselves need to build a new trajectory in their conceptual space. This means that simply attending to a newly found experience might not be enough to incorporate this aspect into one’s cognitive map (the experience just “floats” there without the path that connects it; see Fig. 1a). Such recognized but not-yet-incorporated experiences, in terms of Greenberg and Safran (1989), are just “potentials.” For a potential to become a stable mental representation, the experience needs to be “carried forward” or unfolded (Gendlin, 1969; Stern, 1999) by verbalization and symbolization—something that can be called “conceptualization from experience.” Such conceptualization occurs when a person attends to a raw experiential feeling and tries to express it in words, images, or metaphors (i.e., making meaning of the experience; Lieberman et al., 2007; Torre & Lieberman, 2018). Put differently, when a client is guided into an “unknown” territory, a pathway needs to be built there to make this territory navigable. A representation tied to a certain place in conceptual space allows one to (re)visit this place.

This process allows the raw experience to become a stable mental representation, which in this form can be embedded into the client’s knowledge network, allowing them to operate with it on a higher cognitive level (something that may not be possible with raw experiences). This is precisely why we say that clients should navigate outside their current cognitive maps and create novel concepts beyond. The conceptualization process allows this experience to be embedded into the network via contextualization and meaning-making and thus broadens the internal space that can be navigated for more adaptive reasoning and meaning-making.

In this sense, the meaning-making process is a prerequisite for a coherent integration of new information into the existing mental framework of the client (C. L. Park, 2010). Notably, effective meaning-making by itself entails searching for and expanding the array of information with which a person can operate to obtain a holistic perception of the situation (to explain, e.g., why the situation occurred, its long-term impacts, and its implications; C. L. Park, 2010). Some information may fit the client’s model naturally (leading to assimilation), whereas other information might be needed for the client to be able to process and determine how this new piece of information fits or not (leading to accommodation). In narrative psychotherapy, this process is understood as constructing and revising meaning through the formation of coherent personal narratives that fit clients’ broader understanding of themselves and the world (Gonçalves et al., 2009). Similarly, the meaning maintenance model (Heine et al., 2006) describes meaning as a set of expected associations or relations among concepts, events, and identities. When these associations are disrupted, individuals experience a loss of coherence and engage in compensatory efforts to restore it. Importantly, such restoration does not happen instantly simply because someone provides new information. It requires a gradual process of reflecting on internal contents to integrate new information. Therefore, the expansion of the cognitive map involves meaning-making: Clients must understand how to connect new information to their existing cognitive maps (i.e., beliefs, values, and experiences). Without this process, the insight may remain disconnected and have a limited impact on global behavior or understanding.

### Neural mechanisms of becoming aware

Whereas the previous section explained how the process of becoming aware might work conceptually, we now turn to the neural level. On the neural level, there is less clarity. For example, some of the studies we cite below come from animal models and some from human studies, which might require caution when translating the findings. Thus, in the current absence of an overarching consensus about the presented mechanisms, our primary aim is to provide a generic framework for thinking about it. Therefore, what we present below should be considered one way of looking at a possible neural mechanism of becoming aware. We acknowledge that our ideas might be speculative, and to address this, we provide several testable hypotheses later in the article.

#### Neural mechanisms of mental navigation

Tolman’s concept of cognitive maps obtained even more significance when cognitive maps were discovered in the brain. In seminal rodent neurophysiology studies, “place cells” were found in the hippocampus (O’Keefe & Dostrovsky, 1971), whereas “grid cells” were revealed in the medial entorhinal cortex (Fyhn et al., 2004; Hafting et al., 2005; Sargolini et al., 2006). This research demonstrated that place cells exhibit selective firing when an animal occupies a specific location within an environment, whereas grid cells form a hexagonal grid that covers the space. This phenomenon allows us, for example, to infer an animal’s current location by analyzing the firing patterns of these specialized cells. For instance, if a rat occupies one place, corresponding place cells will fire, and as it moves to a different place, different place cells will activate.

Recent research has started to focus on the potential application of the aforementioned neural mechanisms to spaces that are not physical in nature. Place cells of rodents have demonstrated the ability to encode task-relevant features of abstract concepts such as time, sound, odor, taste, and learned knowledge (Aronov et al., 2017; Eichenbaum et al., 1987; Herzog et al., 2020; MacDonald et al., 2011; Nieh et al., 2021). Similarly, fMRI studies with human subjects have found that the grid-cell system plays a role in encoding visual categories, odor recognition, social hierarchies, word meaning, statistical regularities of events, the structure of complex narratives, concepts in abstract spaces, and semantic relations (Bao et al., 2019; Bicanski & Burgess, 2019; Constantinescu et al., 2016; Garvert et al., 2017; S. A. Park et al., 2020, 2021; Solomon et al., 2019; Theves et al., 2019; Viganò et al., 2021). These findings might collectively indicate that the hippocampal-entorhinal system provides a computational mechanism for encoding and structuring information from different domains, be it spatial orientation or abstract knowledge (Behrens et al., 2018; Bellmund et al., 2018).

This has led researchers to suggest that cognitive maps are used not only for physical-space navigation but also for mental-space navigation (Aru et al., 2023; Behrens et al., 2018; Bellmund et al., 2018). For example, mental navigation within cognitive maps of humans and primates has been shown during decision-making and object-categorization processes (Constantinescu et al., 2016; Neupane et al., 2024), problem-solving (Neupane et al., 2024), and cognitive searches of concepts (Viganò et al., 2023). Viganò and Piazza (2020) showed that the human medial prefrontal cortex and the entorhinal cortex not only encode semantic cognitive maps but also represent the distances and the movement directions between concepts of a novel semantic space. Furthermore, from the neural data, it was possible to reconstruct their relationships in memory.

The findings show that the hippocampus is “blind” to input modality (Aru et al., 2023; Buzsáki & Moser, 2013), leading some researchers to suggest reconceptualizing its function from not just a “GPS system of the brain” for physical space but a generalized mechanism through which we store and navigate memories whether related to spatial, conceptual, emotional, or sensory domains (e.g., sound spaces, problem spaces, valence spaces; Behrens et al., 2018; Bellmund et al., 2018; Buzsáki & Moser, 2013; Eichenbaum, 2017). In this view, the hippocampus is a key structure for episodic, semantic, and relational memory, allowing flexible recombination of past experiences (Eichenbaum, 2017). Thus, its role in navigation is deeply intertwined with memory retrieval and recomposition. This makes the hippocampus especially relevant for psychotherapy, in which the contents of exploration are typically based on either episodic or semantic memory. Fundamentally, mental navigation is a process that operates on memory representations. Schemas are also a type of such representation (Moscovitch et al., 2023); therefore, even if we conceptualize cognitive map expansion and schema change as distinct processes, they likely share a common foundation by relying on hippocampally mediated memory systems.

We suggest that research on mental navigation might, in the future, be extrapolated to study the internal exploration processes within psychotherapy clients. For example, during therapeutic self-exploration, mental navigation means that a trajectory in a neural activity space is traversed—Population A activates Population B, which activates Population C, and so on (Aru et al., 2023). Such sequential firing happens in the hippocampus (Buzsáki, 2015; Buzsáki & Tingley, 2018; Carr et al., 2011; Gupta et al., 2010) and leads to cortical activation (Buzsáki, 1996). Importantly, in the absence of new learning, mental navigation might occur within a limited space, thus reflecting mental-health difficulties (see also Fig. 2). It might be that directing attention and discussing the ideas that were previously not reflected on will lead to the formation of novel (directions within) neural state spaces that can be navigated. To speculate even further, perhaps it would be possible in the future to observe, from the neural data of a client, the structure of the map and changes within it during the course of therapy.

#### Expansion of the cognitive map

A narrow cognitive map, on the neural level, means that navigation occurs only in a restricted area in the neural state space (Fig. 2a). To build up an intuition, consider an example: Activity patterns of neural populations can be described as trajectories in *N*-dimensional neural state space, where *N* corresponds to the activity of individual neurons. A person will arguably visit only a tiny portion of these states in their lifetime (Fig. 2a). In addition, the basic Hebbian principle (Hebb, 1949)—coactivated neurons strengthen their synaptic connections—suggests that the repeated activation of a specific trajectory in this neural activity space will be strengthened every time it is “traversed.” Consequently, once a person “steps” into a given trajectory, the previously reinforced pathways will be activated automatically, guiding the system through the trajectory of least resistance. Without the external help of a psychotherapist, however, overcoming the strength of those previously encoded trajectories may be challenging.

Expanding the cognitive map fundamentally means directing one’s attention and navigating outside of the habitual trajectory (Figs. 2b and 2c). So, instead of letting the client navigate among the preestablished trajectory A-B-C-D-E, a therapist can redirect the client’s attention to some other thoughts A-B-F-G-H, thus revealing a novel place for navigation and for incorporation into a cognitive map (see also Kabrel et al., 2024). The therapist’s role is to lead the client to the unexplored neural activity space; the client’s task is to actively navigate and build a novel trajectory in that direction. Again, this task can entail either (a) expanding neural activity space as it is (i.e., mapping out completely new territories) or (b) switching the navigation process to already existing but underutilized dimensions.

#### Neural mechanisms of concept formation

As pointed out earlier, when a client navigates to the unexplored territory, for it to be integrated into the cognitive map, the pathway or the conceptual representation must become tangible enough to allow the incorporation and the opportunity for future revisiting. In other words, the formation of a stable representation allows the newly explored space to be navigable. Notably, the newly felt experiences in psychotherapy can be concentrated on the immediate present and then fade away, thus remaining at a presymbolic level and unintegrated, or they can be transferred to long-term memory as a stable concept that can be accessed and used later (Moscovitch et al., 2016; Winocur et al., 2010).

In neural terms, the medial temporal lobe memory system is crucial for this integration. For instance, it is known that the hippocampus plays a crucial role in encoding novel memories and concepts (Courellis et al., 2024; De Falco et al., 2016; Rey et al., 2018). Growing evidence suggests that the hippocampal-entorhinal system and medial prefrontal cortex support rapid concept learning by forming multidimensional cognitive maps of relevant features (Morton & Preston, 2021). Furthermore, mental navigation within hippocampal-entorhinal cognitive maps may underlie the imagination of novel concepts (Addis & Schacter, 2012; Buckner, 2010; Morton & Preston, 2021) and achieving insights (Aru et al., 2023; Milivojevic et al., 2015). Relatedly, research in the cognitive neuroscience of insight has shown that moments of sudden representational change are associated with increased activity in the temporal cortex (Becker et al., 2023; Kounios & Beeman, 2014; Tulver et al., 2025). Further conceptual work on insight has also demonstrated how to bind cellular mechanisms such as synaptic plasticity operating within milliseconds to cognitive events such as insight (Aru et al., 2023), thus paving the way for understanding how local neural processes might link to awareness expansion occurring throughout the session or even a series of sessions.

**Fig. 3.**
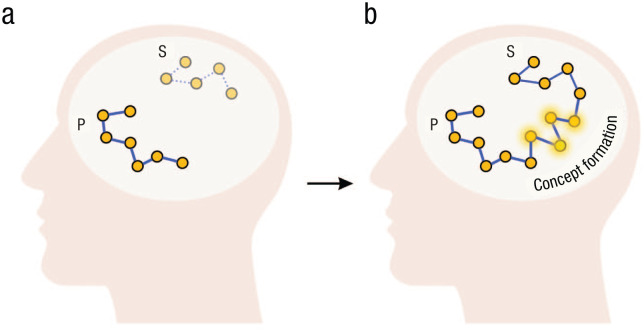
Navigation toward the novel realization. Network P in (a) the narrow cognitive map represents the client’s problem. The network representing the solution (Network S) is currently out of reach of the client’s current understanding (i.e., they cannot come to the realization because it lies several steps away from their current cognitive map). Network S is a direct solution that a therapist gives to the client. In contrast, with the help of a psychotherapist, the client paves the way for connecting the previously limited trajectory with (b) a new trajectory, thus achieving insight. Newly formed concepts (highlighted nodes) could be the concepts the client “maps out” within their cognitive map, led by the therapist’s questions.

Thus, in the therapist’s office, there is an opportunity to conceptualize the experience, associate and embed it into existing knowledge, and consolidate it further through the hippocampal-neocortical “dialogue” (Buzsáki, 1996). Through this process, a newly found concept can be transmitted to long-term memory. We suggest that such incorporation contributes to expansion, which may serve as an internal mechanism facilitating broader cognitive and emotional flexibility (Figs. 2 and 3). That is to say, a novel mental representation emerges in the client’s model of the world, becoming available for further access and elaboration. In several studies, the process of formation of novel concepts was shown to lead to structural changes within the hippocampus, supporting our suggestion (Courellis et al., 2024; Mack et al., 2016, 2018; Milivojevic et al., 2015; Prince et al., 2025). For instance, Milivojevic et al. (2015) showed that achieving insight triggers a systematic reorganization of memory representations and leads to the formation of novel memories in the hippocampus and medial prefrontal cortex.

### Disentangling the thought experiment

Now that we have discussed every component of our framework, we can try to disentangle the thought experiment presented at the beginning of this section (Fig. 1). As a reminder, we introduced this experiment to underscore that if becoming aware is indispensable for the majority of therapeutic modalities, then it is crucial to understand how the process works. Notably, simply providing a solution, without an invitation to (collaboratively) explore deeper how such information relates to the person, might not be enough to trigger a lasting and meaningful realization. Hence, we have argued that clients must engage in mental navigation and pave the way for a novel realization themselves (although with guidance from a therapist) to meaningfully expand their awareness. Although our thought experiment admittedly remains trivial, it shows that the process of becoming aware is far from being trivial. Arguably, providing the client with new information straightforwardly will expand their “factual” knowledge (e.g., Yildirim & Paul, 2024) but not necessarily their “experiential” knowledge (e.g., Borkman, 1976; Paul, 2014). Psychotherapy seeks the expansion of this experiential, immediate, and applied knowledge that clients can rely on to change their thinking, behavior, and, ultimately, lives. It is arguable that, in most cases, only clients’ internally activated mental navigation and the expansion of their understanding from within can bring about the type of awareness that they can act on (see also Moscovitch et al., 2023). This is the hallmark of psychotherapy.

Nevertheless, the question persists: Why can deep and meaningful awareness emerge only through internal exploration? As a response, we suggest that ready-made solutions are effective only when they align with information to which the client can already access internally (for a discussion of schema-congruent learning, see also Moscovitch et al., 2023). To elaborate, individuals can operate with and modify aspects of their cognitive maps only to which they have conscious and voluntary access. When there is no conscious access to certain information within, it becomes challenging for clients to make sense of externally provided solutions related to these inaccessible aspects (because the absence of conscious access makes this aspect “nonexistent” to the individual). Without being aware of the hidden aspects that the therapist is referring to, clients cannot fully grasp or apply the new insights in a meaningful way (Moscovitch et al., 2023). Awareness and understanding of the idea the therapist communicates may emerge only when clients internally connect these previously hidden aspects to their cognitive maps. This is why the therapist needs to guide the client’s internal attention to uncover these hidden places rather than simply providing a ready solution (Fig. 3). The actualization of these places allows further access and the ability to operate with this idea. In other words, a novel aspect becomes integrated into the client’s world model.

We speculate that a successful change through cognitive map expansion would encompass three key domains of everyday human functioning. First, it might facilitate cognitive change by enabling individuals to revise rigid or maladaptive beliefs and to adopt more flexible, nuanced perspectives on the cognitive level. Second, it might result in emotional change by increasing access to underlying feelings and enhancing the ability to regulate emotional responses by better understanding their explicit meanings, goals, and so on. Third, it might mediate interpersonal change by allowing individuals to better understand their role in social dynamics and to modify unhelpful relational patterns as they become increasingly aware of how they contribute to their social relationships (see Fig. 2).

## Compatibility With Other Theories of Therapeutic Change

To construct a metaframework of therapeutic change in dialogical therapies, the framework should not only be applicable to the majority of therapeutic modalities but also explain how it aligns (or not) with the generic mechanisms of therapy suggested previously. We would argue that our framework not only aligns well with some of the existing advanced theories of therapeutic change but also complements them in a meaningful way and fills some of the important gaps. That said, the current framework does not intend to be revolutionary or disruptive: It might admittedly build on or, in places, even overlap with existing principles (see below). However, our aim is to enrich advanced psychotherapy models by offering a perspective from a new angle and with additional nuance, specificity, biological grounding, and practical applicability.

### Reinforcement learning

Reinforcement learning (RL) has long been applied to understand the process of psychotherapy (Krasner, 1961; Truax, 1966; Weitzman, 1967). RL is probably the most suitable framework for understanding the mechanisms of behavioral or exposure therapy (Carey, 2011). However, recent research has started to consider the implications of RL for therapies that are not restricted solely to behavioral interventions (Ludwig et al., 2020; Smith et al., 2020).

For example, in their framework based on RL, Smith et al. (2020) suggested that the strategy that leads people to fulfill their needs is reinforced even if the strategy is suboptimal. Their modeling showed that once a particular strategy has been reinforced, the likelihood that an individual will spontaneously reconsider and adopt a different course of action is very low. This claim is also in line with the work of Craske et al. (2014) on inhibitory learning that posited that therapeutic change occurs by forming new, inhibitory associations that compete with maladaptive ones. Although Smith et al. (2020) remained at the level of abstract computational models, our framework expands on their ideas by offering a neurobiologically plausible mechanism through which such dynamics can be understood and observed. Specifically, what we refer to as a narrow cognitive map reflects the automatic reactivation of previously reinforced strategies. Our model goes further by specifying the therapist’s role: to actively guide clients toward unexplored or underutilized regions of their cognitive map, thereby enabling more flexible and adaptive mental navigation.

Furthermore, Ludwig et al. (2020) also argued that RL can be fruitfully applied to purely mental interventions such as mindfulness-based meditation or cognitive-restructuring techniques. Research has demonstrated that merely focusing on the negative or positive outcomes of a behavior can alter activation in reward and affect-related areas of the brain (Hare et al., 2011; Kruschwitz et al., 2018). Building on this, Ludwig et al. (2020) proposed that as individuals become aware of previously unrecognized and nuanced experiences, their neurocognitive system has the opportunity to update the reward-value calculations (Ludwig et al., 2020). For example, a number of studies have shown that mindfulness training reduces maladaptive eating behaviors by helping people to attend to cravings instead of acting on them (Godfrey et al., 2015; Godsey, 2013; Katterman et al., 2014; O’Reilly et al., 2014).

Similarly, our framework suggests that by expanding the cognitive map people render more and more information accessible to their conscious control; that is, this information becomes mentally reachable. Augmenting previous frameworks, we offer a more precise neural mechanism behind such awareness expansion and suggest how it can be used not only in behavioral practices or mindfulness exercises but also during a therapeutic conversation, implying that therapists can use this mechanism strategically and intentionally. Last, RL suggests a mechanism that unfolds gradually over time, requiring incremental updating of the RL algorithm (Craske et al., 2014; Moscovitch et al., 2023). In contrast, our framework also accounts for more immediate shifts or sudden realizations that can occur in the moment within a flexible therapeutic dialogue without the need for prolonged repetition.

### Predictive processing

A particularly prominent neurocomputational framework known as the free-energy principle or predictive processing (PP; Friston, 2010) has recently been applied to understanding psychotherapy (Chamberlin, 2023; Connolly, 2022; J. Holmes & Nolte, 2019; McParlin et al., 2022; Sheffield et al., 2024; Smith et al., 2020; Villiger, 2025). The PP framework suggests that people construct predictive models of the world based on their past interactions with the environment (A. Clark, 2015; Friston, 2010; Hohwy, 2013). These models are then used to predict how the world works. The PP framework incorporates the probabilities of co-occurrence of certain actions and sensory inputs that are calculated using the Bayesian principles (Friston, 2010). For example, if someone were in a lecture room several times, they would encode a mental representation of this room and expect it to be similar next time. Another important component within PP is prediction error. Prediction error occurs when the expectations (predictions) of the individual are violated (A. Clark, 2015). If a person comes to the lecture room but the furniture is missing, they would experience surprise or prediction error—their predictive model of the world would go through a restructuring to reflect the new reality correctly.

It has been previously suggested that psychotherapy uses various tools to alter the predictions through which clients interpret their problems (Chamberlin, 2023; Holmes & Nolte, 2019; Papalini et al., 2020). For instance, it has been argued that prediction error may underlie exposure (Carey, 2011), therapeutic alliance (Connolly, 2022), and memory modification (Ecker, 2015).

Although both our framework and PP propose that therapeutic change involves integrating new information, there is a difference in emphasis. Our emphasis is less on the mechanism of prediction error minimization and more on the process: how clients are guided to attend to aspects of experience that were previously inaccessible or unformulated. Therefore, rather than being in contradiction with PP, our model contributes to its extension by offering a more process-focused account in the therapeutic context.

Additionally, from a PP perspective, one could argue that any meaningful realization ultimately constitutes a prediction error. We do not dispute this point and agree that there is no fundamental mechanistic split between our framework and PP. However, we focus on a broader range of phenomena, including those that are not experienced as violations or surprises. For instance, in therapy, a client might arrive at a new solution or reframe their experience without ever feeling that their prior expectations were violated. In such cases, the therapist does not trigger a prediction error by provoking conflict but instead facilitates the surfacing of latent information that was already internally stored but not consciously available. We see this distinction as underrepresented in the current PP-based psychotherapy literature, which leans heavily toward emphasizing conflicts with prior expectations (e.g., J. Holmes & Nolte, 2019; Jin et al., 2023; Moscovitch et al., 2023; Sheffield et al., 2024; Villiger, 2025). In contrast, our framework focuses on a different but complementary pathway: gaining additional information about one’s existing internal model without necessarily challenging or contradicting it. Notably, this view is aligned with the computational principles of PP, but it also expands the PP perspectives by showing that representational change in therapy can occur not only through surprise or conflict but also through elaboration and increased granularity of prior models.

Last, whereas PP-based psychotherapy literature typically remains at an abstract computational level of explanation (J. Holmes & Nolte, 2019; Rabeyron, 2022; Sheffield et al., 2024; Villiger, 2025), our framework draws on concrete biologically grounded studies, especially on the hippocampal-entorhinal system, to describe how new concepts and trajectories may be formed and stabilized through mental navigation and concept formation.

### Memory reconsolidation

One theory of therapeutic change that is also based on neuroscientific findings—the memory reconsolidation paradigm—has evoked significant resonance in the field of psychotherapy research (Ecker, 2015; Ecker & Bridges, 2020; Ecker et al., 2012; Lane & Nadel, 2020; Lane, Ryan, et al., 2015). It posits that the mechanism of therapeutic change is similar or analogous to that of memory reconsolidation. For memory to become reconsolidated, it needs to be evoked and then reconsolidated with different meanings and structures. Notably, Ecker (2015) emphasized that novelty is a critical component of this process—memory will not be reconsolidated with a new structure unless new learning occurs, which involves a violation of expectation. Thus, in alignment with the aforementioned PP framework, the schema underlying maladaptive memories must reconsider what it “knows” (Ecker, 2015). In other words, the key feature of memory reconsolidation is the introduction of novel information that challenges and restructures existing memory schemas.

Although the memory reconsolidation framework provides a fundamental mechanism of therapeutic change, it primarily focuses on the end result—memory reconsolidation. However, proponents of this framework often do not elaborate on the importance of internal exploration during memory reconsolidation (Lane, Ryan, et al., 2015). We suggest that this article partially fills this gap and offers a framework for understanding how memory reconsolidation can occur in practice. Moreover, we show why internally directed mental navigation is necessary.

Indeed, a well-cited article on memory reconsolidation in psychotherapy (i.e., “Implicit emotion and emotional trauma”; Lane, Ryan, et al., 2015) stated that “the therapy experience provides new information and that the old memory (or memories) is reconsolidated with this new information” (see also Lane et al., 2022). We seem to converge on this point. Although Lane and colleagues provided a reconsolidation mechanism associated with memory, we build on these ideas to construct a framework that encompasses the process leading to this reconsolidation. Moreover, we argue that awareness expansion comes before such reconsolidation because the newly emerged concepts need to be brought into consciousness and then verbally conceptualized and embedded into the memory network of clients.

### Schema-congruent and -incongruent learning

Another recent framework—schema-congruent and -incongruent learning (SCIL; Moscovitch et al., 2023)—is important to discuss because it has implications for our framework. There are two central components within SCIL. Schema-congruent learning refers to the therapeutic process of encoding information that is consistent with the activated schema (Moscovitch et al., 2023). In contrast, schema-incongruent learning reflects the encoding of the new information that is inconsistent with the activated schema (Moscovitch et al., 2023). Hence, according to Moscovitch et al. (2023), effective schema-change interventions can be understood as a process that consists of weakening the influence of negative, maladaptive schemas and strengthening the impact of positive, adaptive schemas.

Although our frameworks potentially converge on this point, there is also a difference. Although we believe that awareness expansion might involve weakening the influence of negative schemas (e.g., through realizing that a negative self-schema was imposed by someone), we do not emphasize this process as central. The aim of becoming aware is not so much in substituting, for example, the schema “I’m socially incompetent” with “I’m socially competent” but in realizing why a person holds such a belief, what the structure of this schema is, and how a person (unconsciously) contributes to its maintenance (see also Fig. 2). Awareness expansion is not about training positive schemas but about gaining more control of our thoughts and behaviors by realizing more and more information about how they work. Eventually, cognitive map expansion, as we speculate, allows one to become aware of the underlying causes and reasons for the problem and, having mapped out this “territory,” have more opportunities to consciously withdraw from those maladaptive patterns.

Another point in the SCIL model is that schema-incongruent learning should occur through mental simulations—“imitative cognitive constructions of hypothetical events or reconstructions of real events” (Sanna, 2000, p. 168, as cited in Moscovitch et al., 2023). In this way, schema-incongruent learning occurs when a mental simulation violates or challenges the expectation generated by schema, triggering a prediction error and forming new schemas to accommodate these incongruent events (Moscovitch et al., 2023). As pointed out earlier, the incorporation of new information (i.e., cognitive map expansion) may lead to violating experience and thus restructure the schema. Yet, in contrast to SCIL, mental navigation does not necessarily entail mental simulations as defined above. Cognitive map expansion may occur through broader kinds of mental activities; that is, a person does not need to imagine or simulate experiences that are not present—it can be just thoughts, beliefs, arguments, bodily feelings. The person is not expected to self-project in alternative or hypothetical realities (although it still might be a powerful tool for expanding the cognitive map). Rather, a person can decide whether to concentrate on their current conceptual landscape or past/future situations. This can include remembering situations, paying attention to one’s current bodily feelings (e.g., breathing) while describing a given situation or a feeling, or expressing emotions. Each of these activities prompts a person to think of or say things that were not previously in their cognitive landscape, which does not necessarily require mental simulation (Moscovitch et al., 2023). By incorporating these experiences into the conscious part of their mind, they have the opportunity to manipulate this information on higher cognitive levels. This is our central claim: The expansion of the cognitive map allows one to become aware of some information that previously influenced the psychological problem and thus operate with this information more freely. This differs from substituting a negatively valenced cognitive map with a more positive one.

Last, SCIL is based on predictive processing and emphasizes that schema-incongruent learning can occur only in the presence of violating experience. As highlighted earlier, for awareness expansion, the violating experience is not always necessary. What is aimed for is a novel experience, which might not always be violating per se. We suggest that there may be some information that a person has never thought about, but it is not in conflict with the previous information.

However, there are also common denominators between our framework and SCIL. As discussed earlier, memory is one domain in which the underlying computations might overlap because both frameworks rely on the hippocampal system’s role in organizing and accessing memory representations. Yet another important point of convergence is meaning-making. Although SCIL emphasizes schema-incongruent learning through simulation-based prediction errors, and our framework focuses on cognitive map expansion through broader forms of mental exploration, both ultimately rely on the reinterpretation of experience. Meaning-making enables individuals to reorganize memory-based content, be it through imagined scenarios, reflections on bodily states, or the articulation of previously unformulated thoughts. This process allows new insights to be integrated and existing beliefs to be restructured. As discussed earlier, meaning-making may thus serve as a shared component underlying both schema change and awareness expansion.

### Complex dynamical systems

Human cognition and behavior can be fruitfully understood in terms of complex dynamical systems (CDS; Thelen & Smith, 1994). CDS are systems composed of many interacting components that evolve over time, often in nonlinear and unpredictable ways (Thelen & Smith, 1994). Recently, CDS theory has been applied to model the outcome of therapy (Hayes & Andrews, 2020; Heino et al., 2023; Olthof et al., 2023) and can also be augmented by the mental navigation framework.

There are several key components from CDS that are applicable to this article. First, the system’s possible states are represented within a state space that encompasses all potential configurations of the system. Transitions between states may occur because of external perturbations or internal fluctuations (Kelso, 1995). Psychological processes, such as mood regulation or cognitive patterns, can be understood as trajectories within this state space, dynamically shifting in response to internal and external influences. Attractor states within CDS represent stable states or patterns toward which a system tends to evolve over time. Attractors might be shallow, allowing a more flexible transition to another attractor. However, in the context of psychopathology, deep attractors are more relevant. Once these deep attractors are entered, transitions to other states become difficult without substantial external influence (Maiese, 2018; Rolls, 2021). An example of this is chronic depression, with a high risk of recurrence and relapse because of symptom dynamics and complexity (Hölzel et al., 2011). Although such people often experience less intense symptoms of depression than those with a single episode, they tend to constantly return to a depressive state without systematic support, even if they have periods of normal functioning (Hölzel et al., 2011).

In line with the narrow cognitive map, these attractor states represent maladaptive or rigid patterns of thinking and behaving (Maiese, 2018; Rolls, 2021). They are characterized by repetitive loops that keep the individual stuck in their current state, limiting the ability to perceive, process, and integrate new information. By engaging in psychotherapy and integrating new concepts and pathways into their cognitive map, clients may transition from rigid, maladaptive states to more flexible and adaptive configurations (also Hayes & Andrews, 2020). This process might disrupt the existing attractor state by introducing novel information and possibilities that challenge the static pattern. As new concepts and pathways are integrated into the individual’s cognitive map, the system is nudged away from the old attractor state. This shift allows the system to explore new territories of thought and behavior and form new attractor states that are more adaptive and flexible. Importantly, to be able to switch from a pathological attractor, there must be an alternative to switch to (Hayes & Andrews, 2020). Awareness expansion, as presented here, aims to do exactly that. By incorporating more adaptive information into the cognitive map, a person has more options for flexible thoughts and behavior. Furthermore, CDS theory is in line with our suggestion that a simple instruction or insight provided by a therapist might not work. It may be argued that such external intervention cannot cause a perturbation substantial enough to evoke the reconfiguration of parameters within the system.

### Emotional awareness and regulation

Awareness of one’s emotions and, consequently, increased control over them is probably a representative feature of psychotherapy in general. Research shows that various mental-health conditions are related to the decreased ability to regulate one’s emotions (Aldao et al., 2010; Amstadter, 2008; Kring & Werner, 2004) and that emotion regulation has an important role in the process of change (Baer, 2003; Berking et al., 2008; Geller & Srikameswaran, 2015; Lane et al., 2022; Whelton, 2004). Indeed, psychotherapy helps to enhance emotional awareness and regulation irrespective of whether the particular approach is directed at emotional regulation or not (see Gratz et al., 2015). Therefore, there were also claims that the general aim of psychotherapy is to increase the client’s emotional awareness (Burum & Goldfried, 2007; Gratz & Tull, 2010; Greenberg & Safran, 1989; Lane et al., 2022; Pascual-Leone & Greenberg, 2007).

Emotional awareness is particularly important in emotion-focused therapy (EFT; Gendlin, 1969; Greenberg & Korman, 1993; Greenberg & Safran, 1989; Pascual-Leone & Greenberg, 2007). This type of therapy sees becoming aware of one’s emotions as a process of both creation and discovery of implicit elements of one’s experience, such as motor responses and schematic knowledge related to emotion. By attending to and expressing these feelings in words, clients are able to form a stable and organized understanding of their experiences that can be further refined and become more differentiated (Gendlin, 1969; Greenberg & Safran, 1989; Pascual-Leone & Greenberg, 2007). This description of emotional awareness enhancement stems at least from the work of Gendlin (1969) and occupies an important place in many therapeutic modalities. Notably, the current article provides a framework that explains the essential steps of enhancing emotional awareness in more precise neural terms.

First, EFT suggests that emotions are already present, yet they are not actualized in consciousness (for a neural description, see also Solms & Panksepp, 2012). EFT suggests that people need to attend to those feelings and thus become aware of them. We have argued that therapy clients are often unaware of their emotions because they simply do not navigate to their emotional experiences. To become aware of them, a therapist needs to guide the client’s mental navigation process to the emotional experiences instead of rationalizing or avoiding them. Next, EFT claims that emotions need to be put into words and transferred to the conceptual level, at which further higher order level processing becomes possible. We suggest that once a client has reached emotional experiences via mental navigation, they can start talking about them. This conceptualization process, as we speculate, triggers novel concept formation and the creation of novel semantic spaces in the hippocampal-entorhinal system (Viganò & Piazza, 2020), thus transferring vague emotional experiences into stable mental representations integrated into cognitive maps for future navigation. Last, EFT suggests that conceptualization allows the utilization of a new mental representation for reasoning about the problem and constructing more adaptive explanations. We suggest that once a new mental representation is formed, it can be transferred to long-term memory structures and further used for behavioral control and conscious reasoning.

## Cognitive Map Expansion via Mental Navigation as a Metaframework of Therapeutic Change

On the basis of the discussion of different mechanisms of psychotherapy, we propose that the expansion of the cognitive map via mental navigation can be considered a metaframework of therapeutic change applicable to the majority of psychotherapy modalities in which verbal analysis and reflection are at the center of the intervention. By this we mean our framework is flexible and broad enough to incorporate different mechanisms of change that were suggested previously.

As we showed, there are many different mechanisms that can contribute to therapeutic change—memory updating, enhanced emotion regulation, prediction error, schema-incongruent learning, and reinforcement learning (note that we did not mention all possible mechanisms—only the most prominent ones). There are sometimes attempts to explain all (or almost all) cases of therapeutic change through a single unifying mechanism. Memory reconsolidation, for instance, is one such mechanism that was recently suggested to be a unified mechanism of therapeutic action (Ecker, 2024). However, some concerns arise about whether memory reconsolidation really applies to cases beyond those requiring the work with a specific memory (for further concerns, see Moyal et al., 2015). Emotion regulation has also been suggested to be as important, if not more so, than memory reconsolidation (Moyal et al., 2015). Therefore, probably searching for the one and only mechanism of change is a futile and unproductive endeavor. It is possible that the same or different neural mechanisms work for treating the same condition, just as the same or different mechanisms may be effective for treating diverse conditions—there is no simple answer.

Nonetheless, we suggest that becoming aware by mentally navigating cognitive maps, staying on a high level of abstraction, functions as a scaffolding framework for other, more specific theories. Although it may not be definitive on its own, these more specific mechanisms might need a mental navigation process as a prerequisite. To illustrate, common therapeutic concepts such as “restructuring” (or “reorganizing”) a client’s worldview (D. A. Clark, 2014) or “challenging” maladaptive beliefs (Ellis, 1994) are often treated as somewhat distinct pathways to change. However, how do these relate to the idea of cognitive map expansion? Are they separate processes, or do they overlap? We argue that both can be understood as specific instances of the broader metaprocess of cognitive map expansion. Crucially, both reorganization and challenge require the incorporation of new or reinterpreted information. For instance, when a client is challenged, they may come to realize that their previous belief was inaccurate, prompting them to consider alternative perspectives. This, by definition, expands the cognitive map. Similarly, reorganization presupposes that new conceptual content is available (be it completely new information or a new outlook on existing connections), because without it, no restructuring can occur. This view aligns with recent models in the cognitive neuroscience of insight (Tulver et al., 2025) that propose that tension or challenge is a crucial component of insight that introduces instability into existing mental representations, creating a need for novel information to restore coherence, thus facilitating new realizations.

Hence, mental navigation may accommodate multiple mechanisms of change rather than privileging one as the ultimate explanation. Additionally, we hoped to show with our thought experiment that internal exploration is the emblematic feature of psychotherapy that cannot be eliminated. From this follows that the root of psychotherapy’s effectiveness must originate exactly from this component. Although remaining exploratory, we hope this argument will prompt more research into the process of becoming aware.

## Testing the Theory

Although the internal exploration process is essential for enabling transformative realizations, the research on this process has been largely overlooked in psychotherapy. Mental navigation might serve as a framework that can be used to study the process leading to cognitive transformations. We present several hypotheses and methods for testing them below to substantiate or refute the aforementioned suggestions:

Hypothesis 1: *Self-exploration in therapeutic dialogue engages hippocampal-entorhinal cognitive maps, extending prior findings from decision-making and conceptual search to the domain of self-referential processing.*

Previous research has demonstrated that the hippocampal-entorhinal system supports mental navigation during goal-directed decision-making and abstract concept search (e.g., Neupane et al., 2024; Viganò et al., 2023). However, these studies have largely focused on externally oriented tasks involving choices between options, planning paths to goals, or navigating structured semantic spaces. We hypothesize that similar neural mechanisms are recruited during internal self-exploration—specifically when individuals reflect on their life narratives, values, needs, and emotional patterns during therapy. To test this hypothesis, participants could engage in structured therapeutic dialogue tasks inside the fMRI scanner that are designed to evoke internal navigation across autobiographical content. We predict activation of the hippocampal-entorhinal network during these reflective processes. This would suggest that the cognitive map framework generalizes beyond abstract or external problem spaces to include the navigation of knowledge about oneself.

Hypothesis 2: *Increased awareness is associated with the expansion of the cognitive map related to therapeutic content within the hippocampal-entorhinal neural space.*

fMRI studies can probe such changes using mental navigation or cognitive mapping tasks (Constantinescu et al., 2016; Garvert et al., 2017; Viganò et al., 2021, 2023) administered pre- and posttherapy. For instance, participants could navigate conceptual relationships between internal states relevant to therapeutic focus (e.g., reflecting on self-criticism patterns, reasons, causes, etc.). If psychotherapy expands the cognitive maps during mental navigation, this should be reflected in changes in the neural manifold such that previously clustered states become more differentiated, occupy a larger subspace, or are reorganized along novel axes (Heggs et al., 2023; Langdon et al., 2023; Saxena & Cunningham, 2019). This would indicate the activation or transformation of latent neural structures that were previously inaccessible, functionally silent, or encoded in a lower dimensional format and are now engaged along richer conceptual dimensions. If detected, changes in neural activity patterns might also be correlated with various scales of interest such as the Self-Reflection and Insight Scale (SRIS; Grant et al., 2002), Self-Compassion Scale (Raes et al., 2011); clinical outcome questionnaires; as well as the results obtained with the tests outlined below (cf. Hypotheses 3 and 4). However, it remains an empirical question whether new realizations will lead to a literal expansion of the neural activity through changes in the corresponding neural manifold, a restructuring of representational geometry, enhanced functional connectivity, or a more efficient (potentially sparser) coding of relevant information.

Hypothesis 3: *Self-initiated mental navigation, rather than externally provided solutions, leads to restructuring of the cognitive map in conceptual space.*

We predict that only concepts or associations actively inferred by participants during the task will be integrated into the hippocampal-entorhinal representational space. In contrast, decisions or solutions provided by the experimenter spontaneously will not produce a comparable shift in neural patterns. This prediction aligns with our suggestions herein and findings that the formation of novel cognitive maps reflects dimensions that become conceptually important, not just perceptually present (e.g., Garvert et al., 2017). Furthermore, the provision of solutions has shown less effective semantic network changes and enrichment compared with allowing participants to solve problems on their own (Bieth et al., 2024; Herault et al., 2024; Kapur, 2008). Therefore, changes in representational spaces with the suggested intervention can be detected not only by fMRI but also with semantic network modeling—an idea that we unpack in the following hypothesis.

Hypothesis 4: *Cognitive map expansion can be detected as a structural change in an individual’s semantic space.*

Semantic space is understood here as a semantic network in which nodes represent concepts whereas edges denote associations quantified by density (number of edges relative to possible edges) and clustering coefficient (local connectivity; Beaty & Kenett, 2023; Hills, 2024). We predict that people undergoing successful psychotherapy will show increased semantic network complexity posttreatment (e.g., higher connectivity, reduced centrality of maladaptive concepts, more diverse conceptual clustering, and shorter pathways between previously unlinked or distant concepts), and this increase will correlate significantly with increases in self-awareness as measured by the SRIS (Grant et al., 2002). Semantic network modeling (e.g., Beaty & Kenett, 2023; Hills, 2024) is one appropriate method for testing this hypothesis to estimate the structure of a person’s conceptual associations. Pre- and postcomparisons can be conducted using data from free-association tasks, therapy transcripts, or interviews. This method has been repeatedly used in the realm of creativity and problem-solving, bringing valuable insights (for review, see Beaty & Kenett, 2023). For example, the semantic networks of individuals with low creativity are more rigid and disconnected than the semantic networks of individuals with high creativity (Kenett et al., 2014). To our knowledge, there is no research using behavioral/semantic network science in psychotherapy samples, yet we believe the investigation of the clients’ conceptual spaces with semantic networks modeling will bring valuable insights to clinical psychology (for a primer, see Hills, 2024).

## Recommendations for Practicing Psychotherapists

The framework is at the exploratory stage and does not represent a clinical guideline. We have previously provided a set of preliminary recommendations to practitioners using the mental navigation framework (Kabrel et al., 2024). On the basis of our finding that clients used more spatial language (e.g., “I’m going in circles,” “This is unexplored territory,” “I feel like I’m navigating a maze”) during self-exploration in therapy than in other contexts (Kabrel et al., 2024), we proposed that psychotherapists use metaphoric spatial and navigational language more intentionally. Our assumption is that such language might act as a tool to guide mental navigation more efficiently. To elaborate, our findings hinted that self-exploration during psychotherapy feels like a navigation of the internal structure of a psychological problem, which led people to utilize more navigational metaphors to describe this process (see also Bottini & Doeller, 2020). If this is true, then co-constructing these metaphors (Mathieson et al., 2015) and utilizing them to enhance mental navigation appears to be a promising strategy to facilitate moments of becoming aware. For example, to facilitate navigation beyond the narrow cognitive map, a therapist can use phrases such as “You’ve been *navigating* your relationships with [Person X] for a while; how about we try *to go* and look at a more *unexplored territory*. What made you start talking about this today; can you *trace it back*?” or “What you are saying feels like *going in circles*; what if you try to *explore* not their behavior, but your reaction; can you *search for a place* from which all this is coming from?” or “It seems that you don’t want *to go there* [in context of discussing something]; what are the *roadblocks that hold you back*?”

Apart from the linguistic discourse, we believe the concepts of mental navigation and cognitive map expansion can be used without referring to navigational concepts explicitly. It is enough to envisage the client’s exploration process as a navigation within internal conceptual space. If framed like that, perhaps it would be easier to understand the patterns within such navigation (e.g., rigid places where the dwelling time is too long). As our framework suggests, psychotherapists should also ultimately act as a guide or a skilled navigator who helps clients come to new realizations (Kabrel et al., 2024). This includes assisting clients by strategically directing their attention toward novel realization. Simply put, the main question that a therapist should keep in mind is “What should I ask my client so that they will come to this realization?” Hence, the client has to find the way to the novel realization, whereas the therapist has to guide the client to it (i.e., pave the way for the client to “arrive” at this novel understanding).

Furthermore, personalized tools for visualizing the process and progress of psychotherapy are, to our knowledge, not widely used. One could explore whether drawing cognitive maps (for a therapist’s utility or cocreating such maps together with the client) helps to keep track of therapeutic progress. In addition, concept mapping techniques can be used to explicitly draw a problem structure as a narrow map and then draw directions to the expanded map and define what a client should do to expand the map, thus providing a gamification and motivation element to therapy (e.g., a computer game in which a map can be expanded by achieving some skill or knowledge).

Another obvious implication of our framework is that psychotherapists should not provide direct insights to clients, or at the least psychotherapists should ensure that their interpretations and insights make sense (and then clients themselves can elaborate on these insights and embed them into their world model). Sometimes clients can pick up so-called therapy-speak and use it superficially (e.g., “You trigger me,” “I need to defend my boundaries”), which will give them a feeling of insight, whereas in reality it would not give a genuine resolution of a problem. So, real and meaningful insights will entail a personal meaning, extensive elaboration, and generalization to other domains of life than simply those discussed within a particular problem (see also Messer & McWilliams, 2007).

Although some of our recommendations might not seem new, we believe our framework sheds new light on why these recommendations exist and how we can explain their pertinence in more tangible terms.

## Clinical Case Study

To illustrate how the proposed framework might work in practice, consider a hypothetical case of Michael, a 28-year-old man who sought therapy because of a persistent low mood and deep sense of loneliness (for a visualization, see Fig. 2). He often repeats the belief, “No one ever helps me. People just don’t care. I’m always alone.” This conviction shapes much of his emotional state and interpersonal relationships, leaving him feeling disconnected. He meets the criteria for mild to moderate depressive symptoms.

### Initial cognitive map

From the start, it is evident that Michael navigates his internal world along a narrow, habitual trajectory: from interpersonal disappointment to confirmation of his belief that others are unreliable (Fig. 2a). This pattern is emotionally intense, self-reinforcing, and difficult to interrupt. In terms of the cognitive map metaphor, his reasoning circles around a few familiar “locations” (e.g., disappointment, anger, and withdrawal) without adaptive shifts to alternative trajectories.

### Engaging in mental navigation

The therapist introduces the idea of the mind as a network of meanings, beliefs, and memories and describes therapy as a process of internal exploration (see also Kabrel et al., 2024). This psychoeducational metaphor resonates with Michael, who begins to imagine his thoughts and reactions as well-worn paths through a personal terrain. As therapy progresses, the therapist guides Michael to explore areas of this map he rarely visits. For instance, when discussing recent frustrations with a close relative, the therapist asks, “What do you imagine would happen if you asked for help directly?” Michael pauses, then says, “I guess . . . I don’t really ask. I just wait and hope they’ll notice.” At this moment, Michael discovers a new possibility: that his behavior (not just others’) contributes to his sense of isolation (Fig. 2b). Later, deeper exploration brings him to emotionally significant terrain: “When I was a kid, needing something felt dangerous. If I cried, I was ignored or told to stop. I think I learned it’s safer not to need anyone.” This realization connects current patterns to early relational experiences. A new region of the map is now not only discovered but also tied to past and present experience via meaning-making.

### Using navigational metaphors

The therapist returns to the metaphor intentionally and strategically: “It’s like you’ve been walking a single trail that says ‘people don’t help,’ and now you’re mapping out new territory, like the part where you realize you often don’t show when you need something, or that it once felt dangerous to do so.” This helps Michael externalize and reflect on his inner patterns further and consolidate the new realization. As he reaches these new areas and understands how his own behavior contributes to the problem, his interpretation of situations begins to shift. He starts to see that by expressing his needs more clearly or asking for support, he could actively influence outcomes rather than feeling helpless or abandoned. Over time, this growing understanding gives Michael a greater sense of internal coherence and agency. He no longer has to follow the old path that leads to fatalistic or depressive conclusions because he discovered and mapped out new and more effective trajectories.

### Implications within the framework

Michael’s progress illustrates how cognitive map expansion via internal exploration allows for gradual transformation. The familiar belief (“no one ever helps me”) was not simply replaced but expanded and tied to broader contextual implications. New connections emerged: the fear of rejection, the learned avoidance of need, and the desire for connection (Fig. 2c). This process did not rely on a single insight but on iterative movement across conceptual space, facilitated by the therapist’s role in guiding attention to unmapped regions. This case also demonstrates how the navigational metaphor might support psychoeducation and engagement, offering clients an intuitive way to understand their own change process. Note, however, that this example is exploratory and represents a rather idealistic picture of psychotherapy, whereas in reality it might look more complex.

## Critical Remarks

Several clarificatory points should be made before concluding this article. First, although we put an emphasis on the change through internal exploration in a therapeutic dialogue, we should acknowledge that there are cases in which a therapeutic change occurs solely through means that do not require extensive verbal engagement and analysis from the client’s side. For example, such a change might occur through specific interventions directed at behavior change, such as behavioral activation (Ekers et al., 2014) or exposure techniques (Foa & McLean, 2016), or through specific components of psychotherapy such as therapeutic relationships (Wampold & Imel, 2015). However, some researchers argue that even in such interventions the client should engage in self-reflection. For example, during exposure, the client can attend to the arising emotions and thoughts and try to express them (Ludwig et al., 2020). Similarly, Fosha and Yeung (2006) proposed that therapeutic relationships should be reflected on explicitly rather than acting as a hidden factor in the session.

Second, there is a need to address the existence of defense mechanisms and how our framework deals with this concept. The concept of defense mechanisms stems from Freud’s classic psychoanalysis, and it is far from being an overarching concept embraced by every psychotherapeutic modality. In this regard, our framework is more compatible with Stern’s psychoanalytic model (Stern, 1999). The main argument of his work is that unconscious material is not an entity that already exists in a ready-made form simply waiting to be discovered but rather an unformulated, raw experience that must be attended to, reflected on, and articulated to become a differentiated mental representation. In this way, Stern’s work does not align with classic psychoanalysis, which assumes that already formed experiences, for example, memories of traumatic events, are repressed. Instead, Stern argued that we can be unaware of some material not because we refuse to acknowledge it but rather because it has not yet been spelled out. Put differently, “It has not yet attained a form in which consciousness can—or will—grasp it” (Stern, 1999, p. 23). This view also appears to be highly consistent with evidence from contemporary cognitive science. It shows that perceptions are not just unambiguous givens but are constructed through complex neurocomputational processes (A. Clark, 2015; Friston, 2010). Therefore, our framework is conceptualized not in terms of defense mechanisms but rather processing deficits (cf. Lane, Weihs, et al., 2015). Similar to Stern’s view, the concept of “processing deficits” suggests that such conditions as, for example, alexithymia (lack of ability to articulate emotions), are not due to repression but to the absence of a differentiated mental representation tied to an emotional experience (Lane, Weihs, et al., 2015; see also Solms & Panksepp, 2012). Cognitive map expansion, as we have conceptualized it, is an activity that entails working with such raw experiences that are first attended to, then conceptualized, and eventually embedded into a world model as stable mental representations that can be used for coherent thought and behavioral control.

Last, we highlighted in this article the distinction between expanding the cognitive map itself (by introducing and connecting new mental representations to a preexisting cognitive map) and adaptively changing the navigational patterns within already mapped “territories.” This distinction echoes a debate in creativity research between the associative theory, which focuses on the structure of the cognitive network, and the executive theory, which emphasizes how the network is accessed (for a discussion, see Hills, 2024, p. 203). We believe that, as in creativity, both processes are relevant in psychotherapy. Some clients may require transformative realizations that alter the structure of their cognitive map, whereas others may benefit from learning to direct their attention toward more constructive or previously avoided aspects of experience. However, in many cases, both forms of expansion may be necessary as complementary pathways within the same individual. Therefore, a future, more comprehensive model may address a framework that incorporates both types of mental navigation to the same degree, thus providing a fuller account of therapeutic change. Although here we concentrated more on the expansion part, the study of navigational patterns within the client’s internal representational space might present a fruitful new direction for empirical research in clinical samples (e.g., see Hypothesis 4).

## Conclusion

The modern era of psychotherapy began more than 100 years ago with a suggestion that becoming aware of previously unconscious experiences brings positive therapeutic results (Breuer & Freud, 1893; Lane et al., 2022; Lane, Weihs, et al., 2015). Despite the accumulated knowledge and the evolution of psychotherapy practice and science, the general assumption remains largely unchanged to this day. Remaining unrecognized, emotions, drives, convictions provide a basis for maladaptive behavior and decreased well-being. Directing internal attention to these aspects and translating them into concepts turns the experiences into stable, well-differentiated mental representations that can be accessed and built upon for future decision-making, voluntary control, and further conscious elaboration. We have shown that each therapeutic modality and theoretical framework of therapeutic change conceptualizes this process in its own way. Despite this theoretical convergence, the question of how exactly awareness emerges and how the process of becoming aware unfolds remains unanswered.

Here, we attempted to construct a metaframework that would advance our understanding of this question. We have argued that the process of becoming aware would be better understood if framed as a cognitive map expansion via mental navigation. Mental navigation outside the boundaries of previously established and narrow cognitive maps allows one to expand the cognitive map for more adaptive navigation in the future. We hope to have directed the attention of researchers to the importance of the internal exploration process and that future studies will shed a more empirically based light on our suggestions.

## Transparency

*Action Editor:* Zhicheng Lin

*Editor:* Arturo E. Hernandez
